# Visualization of Spirochetes by Labeling Membrane Proteins With Fluorescent Biarsenical Dyes

**DOI:** 10.3389/fcimb.2019.00287

**Published:** 2019-08-20

**Authors:** Chadwick Hillman, Philip E. Stewart, Martin Strnad, Hunter Stone, Tregei Starr, Aaron Carmody, Tyler J. Evans, Valentina Carracoi, Jenny Wachter, Patricia A. Rosa

**Affiliations:** ^1^Laboratory of Bacteriology, Rocky Mountain Laboratories, National Institute of Allergy and Infectious Diseases, National Institutes of Health, Hamilton, MT, United States; ^2^Institute of Parasitology, Biology Centre of the Czech Academy of Sciences, České Budějovice, Czechia; ^3^Faculty of Science, University of South Bohemia in České Budějovice, České Budějovice, Czechia; ^4^Research Technologies Section, Rocky Mountain Laboratories, National Institute of Allergy and Infectious Diseases, National Institutes of Health, Hamilton, MT, United States

**Keywords:** spirochetes, *Borrelia*, *Leptospira*, fluorescent protein, tetracysteine tag, biarsenical dye

## Abstract

Numerous methods exist for fluorescently labeling proteins either as direct fusion proteins (GFP, RFP, YFP, etc.—attached to the protein of interest) or utilizing accessory proteins to produce fluorescence (SNAP-tag, CLIP-tag), but the significant increase in size that these accompanying proteins add may hinder or impede proper protein folding, cellular localization, or oligomerization. Fluorescently labeling proteins with biarsenical dyes, like FlAsH, circumvents this issue by using a short 6-amino acid tetracysteine motif that binds the membrane-permeable dye and allows visualization of living cells. Here, we report the successful adaptation of FlAsH dye for live-cell imaging of two genera of spirochetes, *Leptospira* and *Borrelia*, by labeling inner or outer membrane proteins tagged with tetracysteine motifs. Visualization of labeled spirochetes was possible by fluorescence microscopy and flow cytometry. A subsequent increase in fluorescent signal intensity, including prolonged detection, was achieved by concatenating two copies of the 6-amino acid motif. Overall, we demonstrate several positive attributes of the biarsenical dye system in that the technique is broadly applicable across spirochete genera, the tetracysteine motif is stably retained and does not interfere with protein function throughout the *B. burgdorferi* infectious cycle, and the membrane-permeable nature of the dyes permits fluorescent detection of proteins in different cellular locations without the need for fixation or permeabilization. Using this method, new avenues of investigation into spirochete morphology and motility, previously inaccessible with large fluorescent proteins, can now be explored.

## Introduction

The phylum *Spirochaetes* contains multiple members of medical and veterinary concern, which include *Borrelia* species (Lyme disease and relapsing fever) and pathogenic *Leptospira* species (leptospirosis). Both genera exhibit distinctive morphologies with periplasmic flagella encompassed between inner and outer membranes, which, combined with their unique shape and structure, allow these pathogens to move quickly through tissues during infection. However, the molecular techniques available to identify virulence determinants and key cellular factors are rudimentary in spirochetes compared to those available for some members of the *Enterobacteriaceae*. Such limitations have hampered efforts to identify proteins involved in cell shape, spatial localization of proteins, and transport across the periplasmic space while accommodating flagellar rotation. To address some of the molecular mechanisms underpinning these and other cellular processes, fluorescent proteins and dyes have proven to be valuable tools, permitting detection of spirochete-host interactions (Moriarty et al., 2008; Norman et al., 2008; Dunham-Ems et al., 2009; Bockenstedt et al., 2012; Carrasco et al., 2015; Teixeira et al., 2016; Krishnavajhala et al., 2017), localization or transport of proteins within the bacterial cell (Schulze and Zuckert, 2006; Schulze et al., 2010; Xu et al., 2011; Zhang et al., 2015), or as reporter systems for gene expression (Carroll et al., 2003; Bykowski et al., 2006; Clifton et al., 2006; Eggers et al., 2006; Miller et al., 2006; Gautam et al., 2008, 2009; Whetstine et al., 2009; Aviat et al., 2010; Cerqueira et al., 2011; Grove et al., 2017; Matsunaga and Haake, 2018; Takacs et al., 2018). However, the use of fluorescent protein fusions, most commonly with green or red fluorescent protein (GFP or RFP, respectively), has limitations: the bulkiness of these proteins, typically 25–30 kDa, can lead to atypical protein localization and irregular cellular trafficking (Senf et al., 2008), and some FP alleles have specific pH or oxygen requirements that are not always compatible with the targeted location or underlying biological question. More recent adaptations, such as SNAP- and CLIP-tags, are comparable in size (20 kDA) and require an additional extraneous chemical agent that could impact cell growth and viability in order to fluoresce.

In an attempt to subvert size-related problems caused by fluorescent protein fusions, Griffin and Tsien developed a system for intra- and extra-cellular *in vitro* protein labeling using biarsenical dyes that bind specifically to tetracysteine motifs (Griffin et al., 1998). This method uses a synthetic fluorescent biarsenical compound, such as **Fl**uorescein **A**r**s**enical **H**elix binder (FlAsH) or **Re**sorufin **A**r**s**enical **H**elix binder (ReAsH), which forms a stable complex with a tetracysteine motif consisting of six amino acids (CCPGCC). The central two amino acids of the spacer (proline plus glycine) create a hairpin that reduces steric hindrance between the arsenical groups and the tetracysteine motif, yielding optimum fluorescence (Adams et al., 2002). Using this technique, fluorescent live cells can be viewed in real-time without the issues typically encountered with GFP and other accessory proteins.

Biarsenical dyes have been widely used to study protein dynamics and interactions in various bacteria, viruses and even prions. In an attempt to view components of the Type II secretion system in *Pseudomonas*, Senf et al. initially used GFP fused to their target protein, but the fusion resulted in a non-functional and unstable protein (Senf et al., 2008). In contrast, the nominal mass added by the tetracysteine motif did not perturb the system and allowed successful visualization of the Type II secretion system. Likewise, groups studying viral kinetics encountered similar obstacles with GFP-fusions of polyproteins, which disrupted normal viral function (Panchal et al., 2003; Arhel et al., 2006). However, both groups successfully employed the much smaller tetracysteine tags coupled with biarsenical dyes to visualize HIV infection and Ebola virus assembly. Other examples of the successful use of tetracysteine tags include the visualization of flagellar dynamics in *E. coli* communities (Copeland et al., 2010), *Shigella* effector components of the Type III secretion system entering host cells in real time (Enninga et al., 2005), and converted forms of prion proteins (Gaspersic et al., 2010). More recently, biarsenical dyes have been used to gain an understanding of flagellar elongation and decay in *E. coli* (Zhao et al., 2018). The broad application potential of tetracysteine motifs coupled with biarsenical dyes provides an alternative fluorescent method to visualize cells and proteins when fusion to larger fluorescent proteins may produce aberrant results.

Here, our group utilized tetracysteine motifs and biarsenical dyes to successfully label membrane proteins in live cells of the spirochetes *Leptospira biflexa* and *Borrelia burgdorferi*. We found that the biarsenical dyes can diffuse across the outer membranes of these spirochetes, and that concatenating two copies of the 6-amino acid tag increased the intensity and duration of fluorescence. Mouse-tick infection studies of a tetracysteine-tagged *B. burgdorferi* strain demonstrated that the tetracysteine motif was stably maintained *in vivo* and did not adversely affect infectivity in either the arthropod vector or murine host. Finally, biarsenical-bound proteins could be followed over several days, indicating that this approach can be used in time-course studies of live cells.

## Materials and Methods

### Bacterial Strains and Growth Conditions

All strains used in this study are listed in Table 1. *Borrelia burgdorferi* strain B31 A3 is an infectious clonal derivative of the type strain B31 (Burgdorfer et al., 1982; Elias et al., 2002). *B. burgdorferi* ΔD109, a previously characterized B31 A3 mutant lacking a wild-type copy of the plasmid-borne gene encoding outer-surface protein (Osp) D, was utilized in this study (Stewart et al., 2008). In addition, strain B31 A34, a non-infectious and more readily transformed clone that lacks restriction modification systems (Jewett et al., 2007), was also used. *Borrelia* cultures were grown at 35°C in liquid Barbour-Stoenner-Kelly (BSK)-II medium supplemented with 6% rabbit serum (Pel Freez Biologicals, Rogers, AZ) or in solid BSK medium under 2.5% CO_2_ (Samuels et al., 1994). *Borrelia* growth media were supplemented with antibiotics at the following concentrations when appropriate: gentamicin (40 μg/mL), kanamycin (200 μg/mL), and streptomycin (50 μg/mL).

**Table 1:** Strains used in this study.

**Designation**	**Description**	**Purpose**
*B. burgdorferi* B31 A3	Wild-type infectious (Elias et al., 2002)	Parental strain
*B. burgdorferi* B31 A34	Non-infectious/high-passage (Jewett et al., 2007)	Parental strain
A34-SV-OspDTC	OspD with a single tetracysteine tag expressed from shuttle vector, B31 A34 background (this study). This strain produces both a tagged and wild-type form of OspD	Testing and optimization of FlAsH system in *B. burgdorferi*
A34-SV-OspD2xTC	OspD with a double tetracysteine tag expressed from shuttle vector, B31 A34 background (this study). This strain produces both a tagged and wild-type form of OspD	Evaluating the benefit of concatenating two tetracysteine tags to increase fluorescence and prolong detection
A3ΔOspD 109	*ospD* deletion strain, A3 background (Stewart et al., 2008)	Parental strain lacking *ospD*
ΔOspD-SV-TC	OspD with a single tetracysteine tag expressed from shuttle vector, A3ΔOspD 109 background (this study)	Evaluation of fluorescence and duration of fluorescence in a strain lacking a wild-type version of OspD
ΔOspD-SV-2xTC	OspD with a double tetracysteine tag expressed from shuttle vector, A3ΔOspD 109 background (this study)	For comparing a double tetracysteine tag to a single tag in a strain lacking a wild-type version of OspD; used for OspD re-labeling experiments
A3-LA7TC	LA7 with a single tetracysteine tag created by allelic exchange with the endogenous gene	Used to assess effect of tetracysteine motif on a protein that is important in the *B. burgdorferi* infectious cycle
*L. biflexa* serovar patoc strain Patoc I (Paris)	Wild-type	Parental strain
OmpATC	OmpA with a tetracysteine tag created by integration at the endogenous locus (this study). This strain produces both a tagged and wild-type form of OmpA	Testing FlAsH system in *Leptospira*

*Leptospira biflexa* serovar Patoc (strain Patoc I) was cultured at 30°C in a shaking incubator at 150 RPM in EMJH medium (Fisher Scientific). Solid EMJH media for plating included 1.2% wt/vol Nobel agar (Fisher Scientific), and plates were inverted, sealed with parafilm and incubated at 30°C for up to 1 week. *Leptospira* growth media were supplemented with kanamycin (20 μg/mL) where appropriate.

*Escherichia coli* TOP10 cells (Invitrogen, Carlsbad, CA) were used for recombinant DNA cloning purposes unless otherwise noted. Final antibiotic concentrations were as follows: gentamicin (5 μg/mL), kanamycin (50 μg/mL), and spectinomycin (100 μg/mL).

### Mutant Construction and Transformation

**Figure 1:**
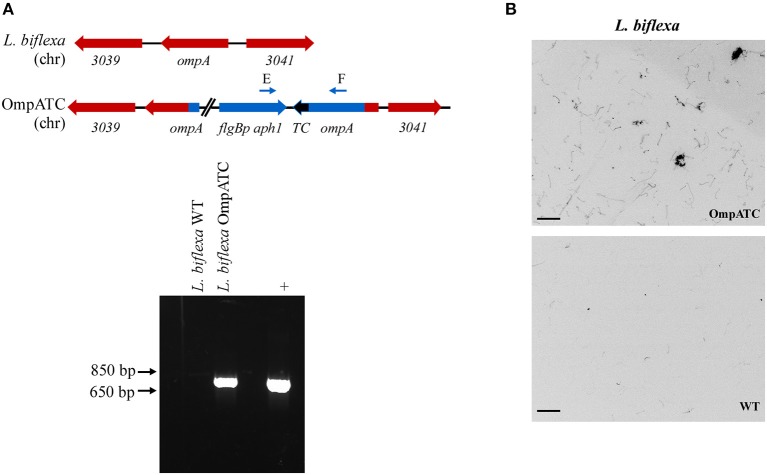
Confirmation of genetically modified and fluorescent *L. biflexa* spirochetes by PCR and microscopy. **(A)** Diagram of relevant genetic loci and corresponding agarose gel image of PCR amplicons. The region in blue denotes the integrated plasmid DNA (plasmid sequences between *ompA* and *aphI* cassette not depicted in diagram), while endogenous loci are depicted in red. Small arrows above diagram indicate oligonucleotides E and F used for PCR confirmation of the *ompATC* integration. Transforming plasmid DNA serves as a positive control (+), while *L. biflexa* WT DNA serves as a negative control. **(B)** *L. biflexa* OmpA fluorescence. FlAsH dye-staining of the tetracysteine-tagged OmpATC strain and WT *L. biflexa* (negative control). Scale bar 10 μm; image inverted.

Oligonucleotides used in mutant construction are listed in Table 2. All plasmids generated in this study were confirmed by sequencing and are listed in Table 3. A tetracysteine motif was added to the 3′ end of a second copy of *ompA* (Outer Membrane Protein A, UniProt accession #: B0SQ62) in *L. biflexa* by targeted integration of a non-replicating plasmid at the endogenous locus. First, the primer pair A/B was used to amplify the *ompA*-like coding sequence and clone it into pGem-T EZ (Promega Inc., Madison, WI). Next, an inverse PCR reaction was used in conjunction with primer pair C/D to add the tetracysteine tag (TC-tag) at the 3′ end of *ompA*, which results in an in-frame addition of the motif to the carboxy-terminus of OmpA. Amplicons were digested, self-ligated, and transformed into *E. coli* Top 10 cells. Next, a kanamycin resistance cassette suitable for use in *L. biflexa* (*flgBp aph1*) was cloned into the construct using the available XhoI restriction enzyme site. Lastly, the ampicillin resistance cassette within pGem-T EZ was inactivated by excising part of the coding region with the restriction enzyme AclI; the plasmid was subsequently self-ligated, transformed into *E. coli* Top 10 cells and selected on kanamycin plates. Colonies resistant to kanamycin were replica-plated for susceptibility to carbenicillin to demonstrate inactivation of the ampicillin resistance cassette. The completed targeted integration construct, pGem::OmpA-TC, was transformed into *L. biflexa* using protocols previously established (Louvel and Picardeau, 2007) and the resulting strain designated OmpATC. Transformants were confirmed by PCR (Figure 1A) and Southern blot analysis (data not shown).

**Table 2:** Primers used in this study.

**Primers**			
	**Name**	**Sequence**	**Function**
**LEPTOSPIRA**			
A	OmpA.SnaBI.For	tacgtaGAGCAG TCCGTTGACAAG	Cloning of *ompA*
B	OmpA.SnaBI.Rev	tacgtaTCG TCTGGTAAGGAT TGG	Cloning of *ompA*
C	iPCR.OmpA.XhoI.For	ctcgagGAAATTCTATTTTCTTACTAGAGACC	Inverse PCR for addition of tetracysteine motif
D	iPCR.OmpA.TC. XhoI.Rev	ctcgagTTAACAACATCCAGGGCAACATTTAGAAACGACTTGGAAAGTCAC	Genetically encoding the tetracysteine motif at the 3′ end of *ompA* before the stop codon (underlined)
E	OmpA.Seq1.For	TAAACTATGGAAACATTAAGGCAGG	Transformant confirmation by PCR
F	Kan.Out.RC	GCAGTTTCATTTGATGCTCG	Transformant confirmation by PCR
**BORRELIA**			
	**OspD1xTC**		
G	PospD-TC.SV2.F	CGGTACCCGGGGATCGGCCATGGGAAGAAGGAG	Amplifying *ospD* and cloning into pKFSSI
H	OspD-TC.SV2.RC	ATGCCTGCAGGTCGATTAACAACATCCAGGGCAACAAGTATTTAACAAGGCCACAACTTC	Genetically encoding the tetracysteine motif at the 3′ end of *ospD* before the stop codon (underlined)
	**OspD2xTC**		
I	OspD.F.SalI	gtcgacCGTCTCTACTGTATTTCCTGC	Cloning of *ospD*
J	OspD.2TC.SalI. Rev	gtcgacTTAGCAACAACCTGGGCAGCAACCTTCATCTCCACAACATCCAGGGC	Genetically encoding the double tetracysteine motif at the 3′ end of *ospD* before the stop codon (underlined)
K	OspD.For	GCTCTCAATATCTTGTGTTC	Transformant confirmation by PCR
	**LA7TC**		
L	LA7.SnaB1.For	tacgtaGCATCAAGTCTTGGTGAATCTG	Cloning of *la7*
M	LA7.SnaB1.Rev	tacgtaCTAGAAATAGACTATGGGCAAGG	Cloning of *la7*
N	iPCR.LA7.XhoI. For	TATTctcgagTTTATATTTTTGATTTTATAGGCTTTAATC	Inverse PCR for addition of tetracysteine motif
O	iPCR.LA7.TC. XhoI.Rev	ATctcgagTTAACAACATCCAGGGCAACAATTCGTTAACATAGGTGAAATTTTTTCAACG	Genetically encoding the tetracysteine motif at the 3′ end of *la7* before the stop codon (underlined)
P	LA7.Seq3. For	GCACGTTTTTCACGCTATG	Transformant confirmation by PCR
Q	Kan736.RC	AAAGCCGTTTCTGTAATGAAGGAG	Transformant confirmation by PCR

*All oligos are shown in the 5′ to 3′ orientation. Restriction enzyme sites are depicted in the primer name and sequences are indicated in lower case. Stop codon sequences are underlined where applicable*.

**Table 3:** Plasmids used in this study.

	**Function**	**References**
**Plasmids**		
pGem-T EZ	Backbone for *ompA* integration construct	Promega
pKFSS1	Shuttle vector for *B. burgdorferi*	Frank et al., 2003
pBSV2G	Shuttle vector for *B. burgdorferi*	Elias et al., 2003
pGem::OmpA-TC	OmpA-TC suicide vector for *L. biflexa*	Current study
pKFFS1::OspD1xTC	OspD-TC shuttle vector	Current study
pBSV2G::OspD2xTC	OspD-2xTC shuttle vector	Current study
pGEM::LA7-TC	LA7-TC allelic exchange vector for *B. burgdorferi*	Current study

The TC-tag was incorporated into the 3′ end of the *ospD* coding sequence by PCR amplification using primers G and H, to create *ospDTC*. This amplicon includes the putative promoter 5′ of the *ospD* gene (Stewart et al., 2008) and 15 bp homologous to the multiple cloning site of shuttle vector pKFSS1 (Frank et al., 2003) The resulting PCR product was cloned into pKFSS1 using the In-fusion kit (Takara, Mountain View, CA), following the manufacturer's recommendations, and transformed into *E. coli* Stellar cells.

The *ospD-2xTC* cassette was cloned into the *B. burgdorferi* shuttle vector pBSV2G (Elias et al., 2003). An amino acid pocket consisting of GDEG was placed between the dual tetracysteine motifs, as described previously (Andresen et al., 2004). Briefly, primer pair I/J was used to PCR- amplify OspD-1xTC and add a second tetracysteine motif at the 3′ end of the PCR amplicon with primer J. PCR products were digested with SalI and ligated into pBSV2G to form the shuttle vector pBSV2G::OspD2xTC.

The coding region of *la7* was replaced with *la7-TC* by allelic replacement using a construct encoding TC-tagged LA7 cloned into pGEM-T EZ. Briefly, primers L and M were used to PCR-amplify *la7*, including ~500 bp upstream and downstream, of *B. burgdorferi* and clone into pGEM-T EZ. Next, the primer pair N/O was used in an inverse PCR reaction to add the tetracysteine motif to the 3′ end of *la7*. Amplicons were digested, self-ligated, and transformed into *E. coli* Top 10 cells. A kanamycin resistance cassette suitable for use in *B. burgdorferi* (*flgBp aph1*) was added to the construct using the available XhoI restriction enzyme site introduced at the previous inverse PCR step. Lastly, ampicillin resistance was inactivated as described above to generate the completed allelic replacement construct, pGEM::LA7-TC.

Allelic exchange and shuttle vector constructs were transformed into *B. burgdorferi* by electroporation as previously described (Samuels et al., 1994). Strains A34-SV-OspDTC and A34-SV-OspD2xTC were created in the high-passaged, non-infectious strain B31 A34, which has a higher transformation frequency than the parental strain B31 A3 and also carries a wild-type copy of the *ospD* gene. Strains ΔOspD-SV-TC and ΔOspD-SV-2xTC were produced by transforming pKFFS1::OspD1xTC or pBSV2G::OspD2xTC into A3 ΔD109, a B31 A3 derivative lacking a wild-type copy of *ospD* (described by Stewart et al., 2008). Both OspD tetracysteine constructs are on shuttle vectors present at multiple copies per cell in *Borrelia*. Note that strain ΔOspD-SV-2xTC produces only an OspD-TC protein, whereas B31 A34-derived strains produce both wild-type OspD (lacking a TC tag) and TC-tagged OspD. In contrast, A3-LA7TC was constructed by an allelic exchange event that replaced the wild-type copy of LA7 on the chromosome in strain B31 A3. All strains were confirmed by restriction enzyme digestion, sequencing, PCR, and immunoblotting. Rabbit anti-OspD antiserum was used at a dilution of 1:1,000 and rabbit anti-LA7 antiserum (kindly provided by Dr. Brian Stevenson) at 1:250.

### FlAsH/ReAsH Assays

Spirochetes were grown to mid-exponential phase (~5 × 10^7^ cells/mL for *B. burgdorferi* and ~5 × 10^8^ cells/mL for *L. biflexa*) and harvested by centrifugation (5,800 RCF for *B. burgdorferi* and 4,100 RCF for *L. biflexa*) for 10 min at room temperature. Pelleted spirochetes were then washed with 1 mL FlAsH wash buffer (50 mM MOPS pH 7.2, 67 mM NaCl, 20 mM NH_4_Cl). Spirochetes were again pelleted and washed in FlAsH wash buffer with 20 mM DTT. After the second wash, pelleted cells were resuspended in 158 μL of FlAsH solution and incubated in the dark at room temperature for 1 h. The FlAsH or ReAsH solution for staining spirochetes was prepared as follows: 2 mM FlAsH-EDT_2_ or 2 mM ReAsH-EDT_2_ (ThermoFisher Scientific) was resuspended in FlAsH wash buffer at a final concentration of 4.75 μM, and DTT was added to a final concentration of 2.5 mM. The solution was then passed through a 0.22 μM filter, and 2 M DTT was added at 1/100 v/v to a final concentration of 22.5 mM. After labeling, 1 mL of FlAsH wash buffer was added and cells were pelleted as described above. Lastly, spirochetes were resuspended in ~50 μL FlAsH wash buffer for visualization.

### Microscopy

All microscopy was done with a Nikon E80i fluorescent microscope. Image files were obtained and analyzed using Nikon Elements version 4.2 and ImageJ software version 2.0.0-rc-69/1.52i. FlAsH dye was observed in the FITC channel while ReAsH was observed in the Texas Red channel. When comparing peak fluorescence, identical settings for exposure and gain were used for all images. Peak fluorescence data were obtained by setting a constant exposure and gain between strains (typically this was an exposure of 1 s with a gain of 9.6x); however, if these settings oversaturated the image sensor, the exposure was lowered for all strains tested. Average peak fluorescence data were obtained by placing a region of interest (ROI) box within a spirochete along an area of uniform fluorescence. The peak fluorescence of that box was calculated with Nikon Elements software and that value represented the peak fluorescence intensity for that spirochete. Approximately 250 spirochetes per group, and 100 measurements of background, pooled from 3 independent biological replicates, were analyzed.

### Flow Cytometry

To assess fluorescence intensity of a larger number of spirochetes, cultures were first FlAsH- or ReAsH-stained, incubated with Hoechst 33342 DNA stain (20 μM) (Thermofisher Scientific) for 30 min at room temperature, and then analyzed with an LSR II BD Flow Cytometer. Spirochetes were gated based on forward scatter (FSC), side scatter (SSC), and FITC. B31 A34 was utilized as a non-fluorescent wild-type control in conjunction with Hoechst 33342 to identify the spirochete populations, while FITC was used to detect the fluorescent spirochete population. B31 A34-derived strains were utilized to avoid introduction of infectious material in the flow cytometer. A34-SV-OspDTC and A34-SV-OspD2xTC were used to determine the effect of concatenating tetracysteine motifs on OspD. FlowJo version 10.4.2 software was used to analyze data and to calculate geometric means of populations.

### Animal Studies

Rocky Mountain Laboratories (RML) is accredited by the International Association for Assessment and Accreditation of Laboratory Animal Care. Protocols for animal experiments were prepared according to the guidelines of the National Institutes of Health and approved by the RML Animal Care and Use Committee. RML mice are an outbred colony of Swiss-Webster mice maintained at Rocky Mountain Laboratories and used exclusively throughout this study. Mice were inoculated with ~5 × 10^3^ spirochetes intraperitoneally and 1 × 10^3^ spirochetes subcutaneously. Infection was assessed by attempted isolation of spirochetes from ear, bladder, and rear ankle joint tissues at 3 weeks post-inoculation.

Larval *Ixodes scapularis* were reared from egg masses laid by engorged female ticks purchased from Oklahoma State University. All ticks were maintained in a temperature- and humidity-controlled chamber (Caron Model 7000-25) at 22°C with 95% relative humidity. Approximately 100 larvae or 10–20 nymphs per mouse were allowed to feed to repletion. A subset of fed nymphs was crushed and the resulting homogenate serially diluted and plated to determine spirochete burden. Fluorescence of FlAsH-stained organisms was assessed immediately for spirochetes derived from mechanically-disrupted, engorged nymph midguts, and from cultured spirochetes isolated from ticks and mouse tissues.

### Time-Course of OspD-Biarsenical Dye Fluorescence

To assess the longevity of FlAsH fluorescence during *in vitro* cultivation, *B. burgdorferi* cells were FlAsH-labeled as described above. Strains lacking a tetracysteine tag were used as negative controls for the procedure. For heat-killed cells, samples were placed at 55°C for 15 min post-FlAsH labeling and observed subsequently for absence of motility; these cells served as a control for passive loss of fluorescence over time. Labeled cells were resuspended in 1–2 mL of BSK-H medium (Sigma) with *Borrelia* antibiotics (20 μg/ml phosphomycin, 50 μg/ml rifampicin, 2.5 μg/ml amphotericin B), placed in cryovials (Corning), and incubated at 22°C in the dark. Spirochetes were counted using Petroff-Hauser chambers to monitor growth, and all cultures were imaged every 48 h for 7–8 days. Experiments were performed as 3 biological replicates and the images shown come from a single experiment representative of the trends observed.

Counter-labeling assays were performed in order to assess FlAsH saturation of OspD protein over time. In cells originally stained with FlAsH where cell concentrations and images had already been obtained for comparison, 400 μL aliquots were removed, and a ReAsH assay performed as stated above.

### Statistics

Statistical analyses were conducted using GraphPad Prism 7 software. Mann-Whitney statistical analysis was applied when comparing fluorescence intensity by microscopy, geometric mean variation by flow cytometry, and spirochete burden in nymphs.

## Results

### Tetracysteine Tags and Biarsenical Dyes to Visualize Spirochetes

As proof of principle, we assessed the use of biarsenical dyes in the model organism *L. biflexa*. Although membrane proteins have not been extensively characterized in this spirochete, a previous study identified a moderately abundant membrane protein, OmpA (Stewart et al., 2016). To assess the functionality of the FlAsH dye technique in *Leptospira*, a tetracysteine tag was added to the carboxy-terminus of OmpA encoded by a second copy of the gene integrated at the endogenous locus (Figure 1A). Transformants were confirmed by PCR utilizing a primer pair spanning the region between *ompA* and the kanamycin resistance cassette (Figure 1A), and transformed spirochetes fluoresced when exposed to FlAsH dye, permitting visualization of the helical shape characteristic of *Leptospira* cells (OmpATC in Figure 1B). In contrast, cells that did not contain a tetracysteine motif (WT negative control, Figure 1B) did not fluoresce when exposed to the biarsenical dye and only minimal background staining was observed.

**Figure 2:**
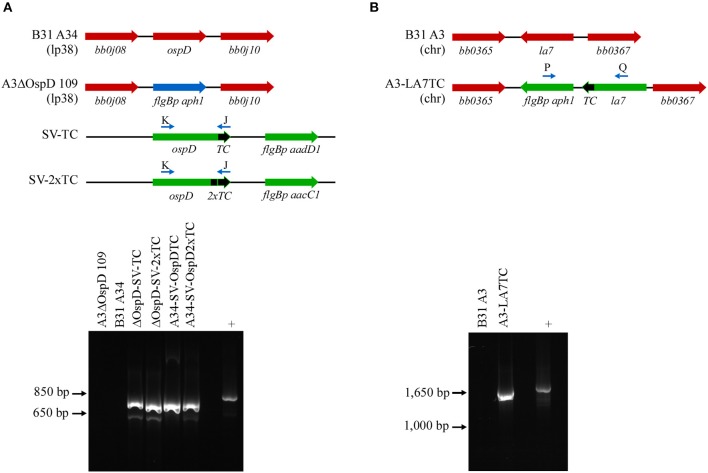
PCR confirmation of genetically modified *B. burgdorferi* spirochetes. **(A)** Diagram of relevant genetic loci and corresponding agarose gel image of PCR amplicons. Native loci are shown in red, the kanamycin-resistance cassette (*flgBp aph1*) is in blue, and relevant portions of the shuttle vectors SV-TC and SV-2xTC are shown in green. Small arrows above diagram indicate oligonucleotides K and J used for PCR confirmation of the *ospDTC* gene in *B. burgdorferi* transformants. The endogenous plasmid (lp38) locus in the parental *B. burgdorferi* strains (A3ΔOspD 109 and B31A34) does not amplify, while the transforming shuttle vector DNA serves as a positive control (+). **(B)** PCR amplification of the chromosomal *la7TC* region utilizing primers P and Q (small arrows above diagram). Native loci are shown in red, while DNA introduced by allelic exchange, including the kanamycin-resistance cassette (*flgBp aph1*), is shown in green. The parental *B. burgdorferi* strain (B31 A3) serves as a negative control, while the transforming plasmid DNA acts as a positive control.

To broaden the utility of this technique, we used a similar approach in another spirochete, *B. burgdorferi*, and tagged both outer and inner membrane proteins OspD and LA7, respectively. These abundant and accessible lipoproteins should be suitable candidates for applying the FlAsH dye technique in *B. burgdorferi*. Spirochetes transformed with a shuttle vector encoding *ospDTC* were confirmed by PCR with primers specific to the *ospD* coding region and the tetracysteine motif; wild-type cells that did not contain the motif did not yield an amplicon (Figure 2A, lower panel). Immunoblot analysis of OspD production in all engineered strains indicated an increase in OspD protein in strains transformed with a shuttle vector encoding either a single tetracysteine motif, A34-SV-OspDTC, or two tetracysteine motifs, A34-SV-OspD2xT (Supplementary Figure 1A). LA7, originally proposed to be an outer membrane lipoprotein (Grewe and Nuske, 1996), but subsequently shown to localize primarily to the inner membrane (von Lackum et al., 2007; Yang et al., 2013), was chosen as a target to assess the ability of biarsenical dyes to freely diffuse across the outer membrane of living *B. burgdorferi* cells. Allelic exchange transformants were screened by PCR using a primer set spanning *la7* and the kanamycin resistance cassette (Figure 2B, lower panel), and spirochetes containing a tetracysteine motif engineered on LA7 (strain A3-LA7TC) produced comparable levels of protein as their wild-type counterpart (Supplementary Figure 1B).

**Figure 3:**
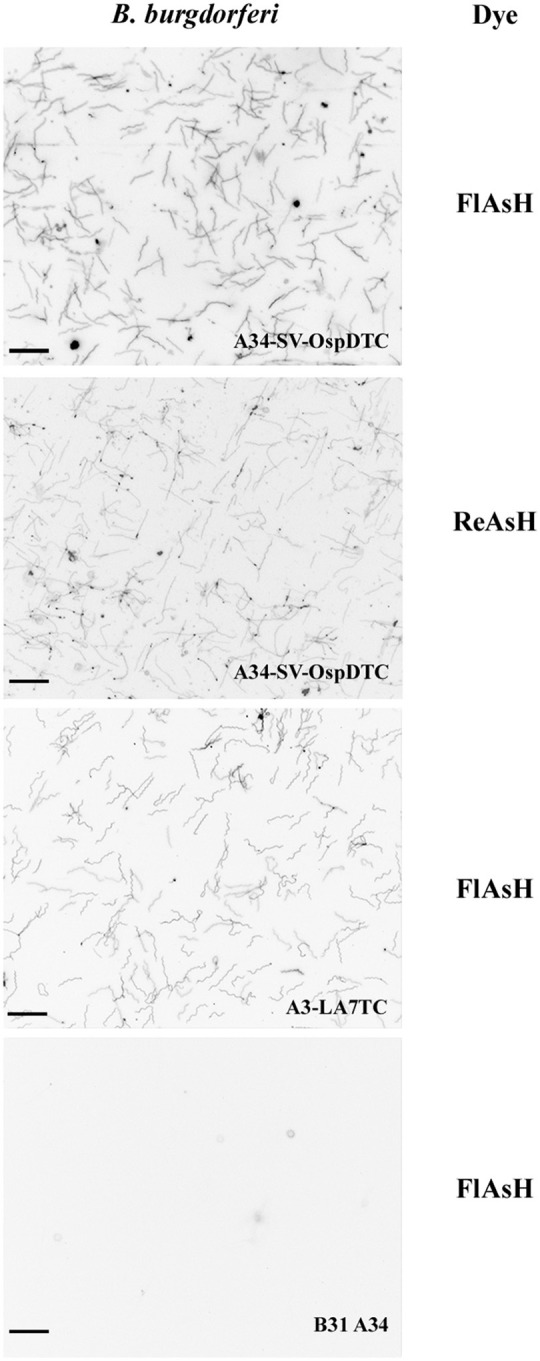
Biarsenical dyes effectively label *B. burgdorferi* with outer and inner membrane proteins containing tetracysteine (TC) motifs. Spirochetes bearing TC-tagged outer surface protein D (A34-SV-OspDTC) fluoresce when exposed to either FlAsH (top panel) or ReAsH (second panel from top). Spirochetes synthesizing TC-tagged inner membrane protein LA7 (A3-LA7TC) fluoresce when exposed to FlAsH (second panel from bottom). In contrast, B31 A34 wild-type *B. burgdorferi* (negative control, bottom panel) does not fluoresce when exposed to FlAsH dye. Scale bar 10 μm; images inverted. All images were acquired using the same settings.

After confirming the presence of the tetracysteine motif in all strains, we next assessed the FlAsH system in *B. burgdorferi*. Fluorescent spirochetes were observed when the tagged strains A34-SV-OspDTC and A3-LA7TC were exposed to FlAsH or ReAsH dye, whereas WT B31 A34 spirochetes (negative control) displayed only background fluorescence (Figure 3). This demonstrated successful application of the technique to inner and outer membrane proteins of *B. burgdorferi*, and thus confirms that biarsenical dyes can diffuse across the outer membrane of live spirochetes without the need for fixation or permeabilization procedures.

### Optimization of the FlAsH Technique

**Figure 4:**
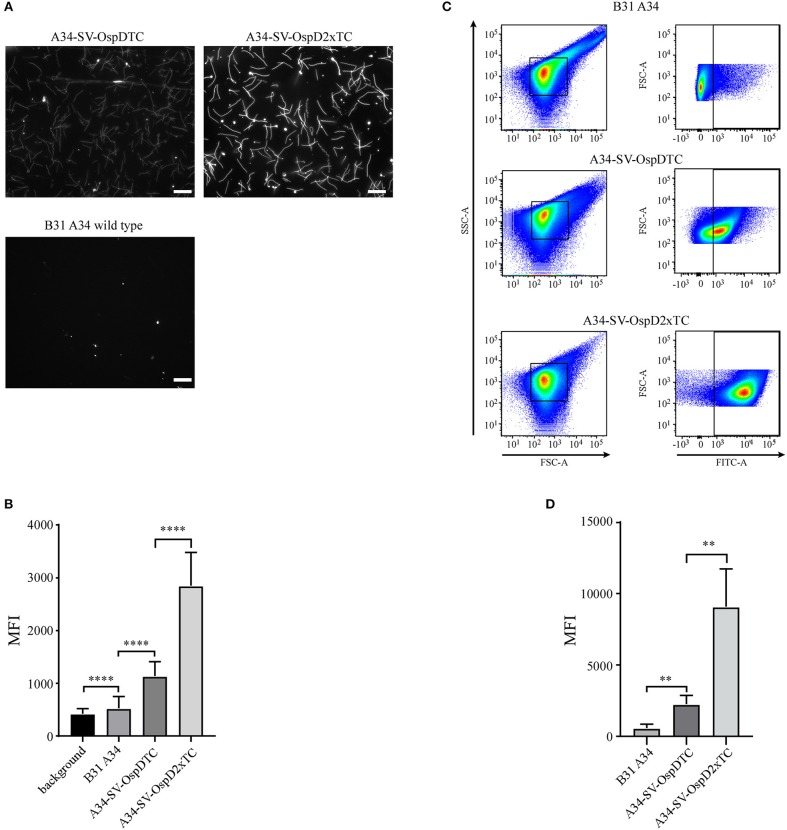
Optimization and quantification of the FlAsH dye system for *B. burgdorferi* membrane proteins containing tetracysteine (TC) motifs. **(A)** Spirochetes bearing OspD with two tetracysteine motifs fluoresce more brightly than those carrying OspD with a single TC tag. Unmodified *B. burgdorferi* strain B31 A34 does not fluoresce in the presence of FlAsH dye, but addition of a single tetracysteine-tag to OspD (A34-SV-OspDTC) allows FlAsH binding and fluorescence. Addition of a second tetracysteine motif to OspD (A34-SV-OspD2xTC) further increases fluorescence. All images were captured with identical exposure times and not inverted. Scale bar = 10 μm. **(B)** Quantification of differences in relative fluorescence of *B. burgdorferi* strains by microscopy. Addition of a second tetracysteine motif on OspD significantly increased fluorescence intensity (A34-SV-OspDTC 1,136 ± 272.3 MFI vs. A34-SV-OspD2xTC 2,850 ± 628.5 MFI, Mann-Whitney test *p* < 0.0001). Background in areas where there were no spirochetes, as well as of *B. burgdorferi* cells lacking tetracysteine motifs, were 421.1 ± 95.95 MFI and 524 ± 225.6 MFI, respectively. Individual spirochete fluorescence was quantified using Nikon Elements software version 4.2 and compiled to generate an average fluorescence per strain. Mean Fluorescence Intensity = MFI. Standard deviation is depicted by error bars (****) *P* < 0.0001. **(C)** Detection and differentiation of FlAsH-labeled spirochetes containing a single or double tetracysteine motif by flow cytometry. Addition of a second tetracysteine motif on OspD significantly increased fluorescence intensity (geometric mean for A34-SV-OspDTC 2,247 ± 603.5 MFI vs. A34-SV-OspD2xTC 9,079 ± 2,648 MFI, *p* < 0.0079). FSC-A, forward scatter; SSC-A, side-scatter; FITC-A, measure of fluorescence-intensity. **(D)** Quantification of the geometric mean data from 5 independent flow cytometer runs analyzed in Prism for statistical significance. Mean Fluorescence Intensity = MFI. Standard deviation is depicted by the error bars (**) *P* < 0.008.

Having ensured that biarsenical dyes could be utilized in spirochetes, we next assessed whether adding a second tetracysteine tag would augment fluorescence. Using identical imaging conditions between B31 A34, A34-SV-OspDTC, and A34-SV-OspD2xTC, a significant increase in fluorescence intensity was observed with addition of a second tetracysteine motif onto OspD (Figures 4A,B), while the overall OspD protein level appeared similar between strains A34-SV-OspDTC and A34-SV-OspD2xTC (Supplementary Figures 1A–D), indicating the increase in fluorescence was a result of the second tetracysteine motif. FlAsH dye-labeled OspD2xTC spirochetes retained motility and grew comparably to unlabeled organisms when incubated at 35 degrees, confirming their viability and the lack of toxicity of the protocol. Also, the addition of a second tetracysteine motif allowed prolonged detection of the fluorescent signal (see Supplementary Figure 2).

FlAsH-labeled spirochetes were also analyzed by flow cytometry to quantify and compare fluorescence of a larger number of cells (Figure 4C). Using this method, we independently validated that two tetracysteine tags fused to OspD resulted in a significant increase in mean fluorescence relative to cells containing OspD with a single tag (Figure 4D). Hence the relative mean fluorescence intensity of spirochetes measured by both methods, fluorescence microscopy and flow cytometry, were in agreement for these *B. burgdorferi* strains.

Surprisingly, while optimizing the protocol, we found that FlAsH dye would non-specifically bind to heat-killed *B. burgdorferi* cells in the absence of tetracysteine motifs (Supplementary Figure 3). The nature of this non-specific interaction between the dye and spirochetes lacking an engineered tetracysteine pocket is not clear. However, this fluorescence requires the FlAsH dye and is only detected in the FITC channel, indicating that it does not reflect general autofluorescence of dead spirochetes. It should be noted that although non-specific staining of heat-killed spirochetes could be abrogated by increasing the concentration of DTT, there was a concomitant decrease in the fluorescence intensity of viable spirochetes containing tetracysteine motifs (data not shown). Hence there is a balance to be achieved between the concentration of reducing agent and fluorescence intensity. Taking these observations into consideration, 20 mM DTT was chosen as the optimal concentration, and WT spirochetes included as negative controls in all experiments to confirm specificity of labeling.

### Tetracysteine Motifs Are Stable Throughout the Mouse-Tick Infectious Cycle

In nature, *B. burgdorferi* cycles between tick vectors and vertebrate hosts. Potentially, addition of the tetracysteine tag to a protein such as LA7, which is important during tick acquisition (Pal et al., 2008), might alter infectivity or destabilize the target transcript or protein *in vivo*. To examine this possibility, we needle-inoculated mice with either wild-type A3 or the A3-LA7TC strain. All mice became infected (5 out of 5 mice for each strain), and both strains could subsequently be re-isolated from mouse tissues (ankle joint, bladder, and ear).

**Figure 5:**
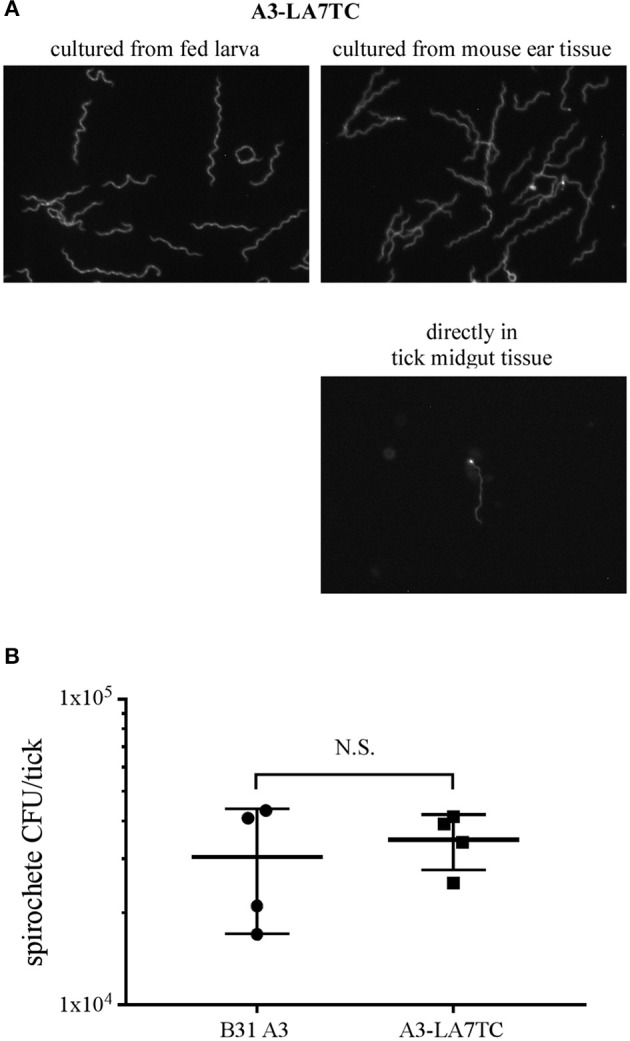
*B. burgdorferi* strain with tetracysteine-tagged inner membrane protein LA7 can colonize and be transmitted between the tick vector and murine host. **(A)** FlAsH-labeling of A3-LA7TC after isolation from tick larvae that fed on infected mice (left), and from ear tissue of mice fed on by infected nymphs (right). Spirochete labeled directly in midgut tissue dissected from an engorged nymph (lower panel). Images have been cropped and are representative of what was observed. **(B)** Strain with tetracysteine-tagged LA7 (A3-LA7TC) colonizes ticks as efficiently as WT (B31 A3). Spirochete burden was determined as CFU in fed infected nymphs by individually macerating and plating 4 replete ticks per group after feeding on naïve mice. The mean and standard deviation bars are shown. No significant difference was detected between strains using the Mann-Whitney test. Standard deviation is represented by the error bars (N.S. = Not Significant) *P* < 0.89.

Before the A3-LA7TC-infected mice were euthanized for attempted spirochete isolation, they were fed upon by naïve *I. scapularis* larvae. A subset of replete larvae was mechanically disrupted and the resulting homogenates cultured to evaluate acquisition of *B. burgdorferi* and whether spirochetes retained the tetracysteine tag. Among larvae that fed on wild-type A3 or A3-LA7TC-infected mice, 4/5 and 5/5 larvae, respectively, acquired spirochetes. Further, upon addition of FlAsH dye, all A3-LA7TC spirochetes isolated from larval ticks fluoresced (Figure 5A, top-left panel), indicating retention of the tetracysteine motif, while A3 spirochetes did not fluoresce (Supplementary Figure 4).

After molting to the nymphal stage, a subset of unfed ticks were mechanically disrupted and cultured to gauge the transstadial retention of *B. burgdorferi*. Of these, all of the A3-LA7TC -infected nymphs (5 out of 5) and 80% of the A3-infected nymphs (4 out of 5) maintained *B. burgdorferi* through the molt, and spirochetes cultured from the A3-LA7TC outgrowths were fluorescent when stained with FlAsH dye (data not shown).

Lastly, transmission was assessed by allowing the remaining infected nymphs to feed on naïve mice. All naïve mice fed upon by infected nymphs acquired *B. burgdorferi* (5 out of 5), as verified by the presence of spirochetes in tissue outgrowths; when spirochetes in a subset of those outgrowths were subjected to FlAsH assays, A3-LA7TC spirochetes were fluorescent (Figure 5A top-right panel). In addition, although technically challenging due to the absolute number of spirochetes in fed nymph midguts, it was possible to detect fluorescent spirochetes directly from A3-LA7TC infected, engorged nymphs that had been mechanically-disrupted and FlAsH-labeled (Figure 5A, lower right panel). Replete ticks were collected and a subset (4 per strain) was used to estimate the number of spirochetes per tick. On average, there was no significant difference in spirochete burden between A3- and A3-LA7TC-infected ticks (3.04 × 10^4^ vs. 3.48 × 10^4^ spirochetes per tick, respectively; *p* = 0.89; Figure 5B). Taken together, the overall infection and fluorescence data demonstrate that spirochetes bearing a tetracysteine motif on LA7 exhibit a WT phenotype throughout the mouse-tick-mouse infectious cycle and can be FlAsH-labeled either directly in tick midgut tissue or after *in vitro* cultivation of spirochetes from tick or murine tissues.

### FlAsH-labeled Proteins Retain Fluorescence Over Time

**Figure 6:**
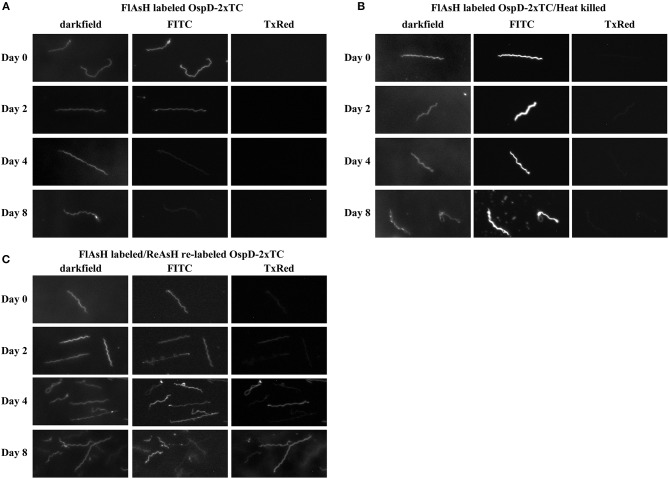
Spirochetes making tetracysteine-tagged OspD can be labeled with FlAsH and relabeled with ReAsH over time. All samples were imaged by dark field and fluorescence microscopy using FITC and Texas Red channels. **(A)** FlAsH-labeled ΔOspD-SV-2xTC fluorescence monitored in the FITC channel over time. No fluorescence was observed in the Texas Red channel (TxRed). **(B)** Fluorescence of FlAsH-labeled and heat-killed ΔOspD-SV-2xTC (control) visualized over time, no fluorescence was observed in the Texas Red channel. **(C)** Fluorescence of ΔOspD-SV-2xTC spirochetes labeled with FlAsH (monitored in the FITC channel) on Day 0 and relabeled with ReAsH (monitored in the TxRed channel) at subsequent time points as indicated. All cultures were incubated at 22°C and analyzed over 8 days. All images were acquired using a 1 s exposure for FlAsH and a 2 s exposure for ReAsH. Images have been cropped, are not inverted, and representative of what was observed. This experiment was repeated 3 times.

We utilized biarsenical dyes to assess the length of time FlAsH-labeled protein could be detected during *in vitro* cultivation. As previously described, we complemented strain A3 ΔOspD in trans with a double tetracysteine-tagged copy of *ospD* on a shuttle vector (ΔOspD-SV-2xTC). We used this complemented strain in time-course experiments in order to eliminate potential competition with WT OspD and have only the tetracysteine-tagged OspD variant available for processing and insertion into the outer membrane. ΔOspD-SV-2xTC spirochetes were pelleted, labeled with FlAsH dye, and then washed and resuspended in BSK culture medium. When fluorescence was monitored during subsequent *in vitro* growth at 35°C, the signal diminished almost entirely after 24 h (data not shown). Presumably this observed loss of fluorescence was due to dilution of FlAsH-labeled OspD by growth and cell division that occurred within this timeframe (~5 doublings and ~30-fold increase in cell number). Therefore, we performed this experiment at 22°C, a condition in which *Borrelia* grows more slowly, to reduce the dilution of labeled protein resulting from cell growth and division. Fluorescence of the ΔOspD-SV-2xTC strain was monitored over the course of 8 days (Figure 6A). Spirochetes of the same strain that were FlAsH-labeled and then heat-killed were included as a metabolically inert control that does not undergo cell division or have an active mechanism for removing or shedding protein (Figure 6B). ΔOspD-SV-2xTC fluorescence diminished over the time course, compared to day 0, but remained detectable. Passive loss of fluorescence of the FlAsH signal did not appear to be an issue, as fluorescence was relatively stable throughout the time course for the heat-killed samples.

At each time point, we relabeled the cells with ReAsH, a red variant of biarsenical dyes (Figure 6C). This pulse-chase style experiment allowed visualization of cells that bound both FlAsH and ReAsH over time. At day 0, very little ReAsH was taken up by cells, whereas the ReAsH signal grew appreciably by days 4 and 8, These data suggest that binding sites for ReAsH became available as FlAsH-labeled OspDTC waned and unlabeled OspDTC increased in the spirochete membrane over time.

### Discussion

In this study we describe the application of a new technique in spirochetes for fluorescently labeling specific proteins using tetracysteine tags and biarsenical dyes. This technique allowed fluorescent labeling of spirochetes by incorporating a 6 amino acid motif at the C-terminus of both inner or outer membrane proteins, and staining with a membrane-permeable biarsenical dye that binds to this motif. The small increase in protein size conferred by the tetracysteine motif minimizes potential negative effects that may occur when targets are fused to larger fluorogenic proteins (e.g., GFP or RFP), such as steric hindrance, causing improper protein folding, trafficking to the incorrect cellular location, or inappropriate oligomerization. We successfully applied the same protocol to the distantly related spirochetes *B. burgdorferi*, a zoonotic human pathogen, and *L. biflexa*, a free-living saprophyte, suggesting that the FlAsH system can be applied broadly across all spirochete species.

This technique worked well for both the outer membrane protein OspD and the inner membrane protein LA7 of *B. burgdorferi*, indicating that the dye is freely diffusible across the intact outer membrane of living spirochetes without the need for fixation or permeabilization. Although the membrane location of OmpA in *L. biflexa* has not been definitively verified, it is predicted to possess a lipoprotein signal peptide by LipoP 1.0 (http://www.cbs.dtu.dk/services/LipoP/) and was identified in the membrane-associated protein fraction of leptospires (Stewart et al., 2016). Further, *ompA-TC* and *la7-TC* constructs were targeted to their respective endogenous loci, while the *ospD-TC* fusions were expressed *in trans* from shuttle vectors, indicating that single copy (endogenous locus) or multicopy (shuttle vector) expression sites are both effective options.

As with any technique, the FlAsH system has limitations and required some optimization. Biarsenical dyes rely on precisely spaced and oriented thiol groups to bind and fluoresce. However, due to the cellular abundance of protein thiols, non-specific binding can occur and occlude proper protein visualization. This can be remedied by reducing protein disulfide bonds in the cellular environment before labeling with dithiol compounds such as 1,4-dithiothreitol (DTT) or 2,3-dimercaptopropanol (BAL). Unexpectedly, we noticed that heat-killed, wild-type spirochetes will non-specifically bind the FlAsH dye. It does not appear to be general autofluorescence of dead spirochetes because the fluorescent signal is only detectable in the FITC channel, not the Texas Red, or DAPI channels, and requires the FlAsH dye. In an attempt to alleviate this issue, we increased the DTT levels 10-fold, which lowered fluorescence in both viable and dead spirochetes. We speculate that heat-killed spirochetes have exposed di-sulfide bonds that are otherwise inaccessible, resulting in the observed binding of the biarsenical dye.

Biarsenical dyes photo-bleach faster than fluorescent proteins, and the fluorescent signal of a tetracysteine-tagged protein is typically several fold lower than that of a cognate GFP fusion (Adams et al., 2002; Crivat et al., 2011). Also, proteins less abundant than OmpA, OspD, or LA7 have not been tested. However, these limitations could potentially be mitigated by adding a dual tetracysteine motif to the protein of interest, which not only increases signal intensity, but also prolongs fluorescence. HeLa cells transiently transfected with a tetracysteine motif on α-tubulin indicated a positive linear relationship between additional tetracysteine tags and fluorescence intensity (Van Engelenburg et al., 2010). Our results with OspD-TC in *B. burgdorferi* agree with this finding.

We also noted loss of fluorescence within 24 h when FlAsH-labeled spirochetes were cultured at 35°C, presumably due to cell growth and division, where segregation of labeled protein to daughter cells combined with synthesis of new protein leads to dilution of the signal on individual bacteria. We are not aware of similar studies examining the duration of fluorescent protein fusions in *B. burgdorferi* as a comparator. However, when grown at 22°C, *B. burgdorferi* replication is significantly slower and fluorescence could be monitored over a longer time frame. At this temperature, we were able to follow FlAsH dye-stained OspD on the surface of *B. burgdorferi* for 8 days, albeit with diminishing signal. Re-labeling assays (using the red-variant, ReAsH) indicated that there was very little OspD available to re-label at the onset of the experiment, suggesting that the initial dye labeling was highly efficient. With time, OspD protein became available for re-labeling and we observed increased fluorescence with ReAsH. This is in contrast to spirochetes that were heat-killed and do not lose fluorescence over time. Together, this would suggest that the decrease in FlAsH fluorescence with time in culture is not due to diffusion of the dye into the media, but to cell division and new protein synthesis, leading to dilution of the labeled protein in the daughter cells. However, another possible explanation for loss of fluorescence could be due to shedding of labeled protein into the media via membrane bound vesicles, or active release by an undefined mechanism.

A significant attribute of the biarsenical dye system is the small size of the tetracysteine motif, which does not impede critical protein function *in vivo*. Previous work on LA7 indicated a role in tick acquisition, with upregulation of expression of the gene encoding LA7 (*bb0365*) in feeding ticks (Pal et al., 2008). LA7-deficient spirochetes were severely impaired in both tick acquisition and transmission. Here, we show that a strain with an engineered tetracysteine motif on LA7 is fully competent in an experimental mouse-tick-mouse infectious cycle. No difference in the spirochete burden per nymphal tick was observed between TC-tagged and WT strains. When subjected to FlAsH assays, TC-tagged spirochetes isolated from mouse tissues or ticks fluoresced, indicating retention of the tetracysteine motif on the protein. A further advantage of TC-tagged spirochetes is the ability to directly visualize live spirochetes in dissected tick tissues, allowing assessment of target gene expression without fixation or culturing. Together, these data demonstrate that the gene encoding a TC-tagged protein is stably maintained throughout the infectious cycle and that addition of the TC tag does not interfere with the function of a protein that is important for maintenance in the tick. An additional benefit of this system over other fluorogenic methods includes the freely diffusible nature of biarsenical dyes, allowing labeling of sub-surface proteins in live spirochetes without fixation or permeabilization.

We demonstrate that tetracysteine motifs coupled with biarsenical dyes are capable of labeling abundant membrane proteins in both *B. burgdorferi* and *L. biflexa*, and likely can be extended to proteins in other cellular locations in these and other spirochetes. Compared to their fluorescent protein counterparts, the small size of the tetracysteine motifs makes them ideal for protein localization and trafficking studies. Genetically encoded tetracysteine motifs coupled with biarsenical dyes represent a novel molecular tool for studying spirochetes, and can provide valuable insights into spirochete morphology, protein-protein interactions, and cellular trafficking.

## Data Availability

All datasets generated for this study are included in the manuscript/Supplementary Files.

## Author Contributions

PS conceived and supervised the study with input from PR. CH, HS, MS, and TE contributed to the design and conducted the FlAsH dye experiments. VC and JW assisted with the animal study. AC analyzed samples by flow cytometry. TS provided advice and assistance with image analyses. CH took the lead role in manuscript preparation with direction and assistance from PS and PR.

### Conflict of Interest Statement

The authors declare that the research was conducted in the absence of any commercial or financial relationships that could be construed as a potential conflict of interest.

## Supplementary Material

Supplementary Figure 1 OspD and LA7 protein levels in B31 strains. **(A)** Immunoblot analysis using antisera recognizing OspD (~28 kDa). OspD protein levels are comparable in strains transformed with SV-OspDTC and SV-OspD2xTC **(B)** Immunoblot analysis using antisera recognizing LA7 (~22 kDa). LA7 protein level is comparable between strains B31 A3 and A3-LA7TC. **(C,D)** Coomassie brilliant blue-stained polyacrylamide gel indicating relative protein loads of samples used for immunoblots in **(A,B)**, respectively.

Supplementary Figure 2 Duration of fluorescence increased by the addition of a second tetracysteine motif. Fluorescence of strain A34-SV-OspDTC was compared to A34-SV-OspD2xTC over time. Videos were taken with Nikon Elements software and data analyzed in Prism. Measurements were taken of three spirochetes/strain during the same experiment. Fluorescence represents the intensity of spirochetes measured with the Nikon Elements software; time measured in seconds (s).

Supplementary Figure 3 Non-specific binding of FlAsH dye to heat-killed spirochetes. A3 spirochetes that do not contain a tetracysteine motif, but were heat killed prior to FlAsH staining, readily take up the dye and fluoresce (1st row) similarly to A3-LA7TC spirochetes containing a tetracysteine motif (2nd row). Spirochetes that have been heat-killed but not incubated with FlAsH, do not fluoresce (3rd and 4th row). Fluorescent spirochetes are only detected in the FITC channel, not in any other channels (not shown).

Supplementary Figure 4 *B. burgdorferi* wild-type spirochetes (B31 A3) isolated from tick vectors and murine hosts do not fluoresce after labeling. FlAsH-labeling of spirochetes isolated from larval ticks fed on B31 A3-infected mice **(top)**; spirochetes isolated from ear tissue of mice fed on by B31-A3 infected nymphs **(middle)**; and spirochete in dissected midgut of an engorged B31 A3-infected nymph **(bottom)**. Representative dark field and FITC images are shown.
