# Supplementary figures and images for: Visualization of Spirochetes by Labeling Membrane Proteins With Fluorescent Biarsenical Dyes
Source: Front Cell Infect Microbiol. 2019 Aug 20;9:287. doi: 10.3389/fcimb.2019.00287 (PMC6710359; doi:10.3389/fcimb.2019.00287)

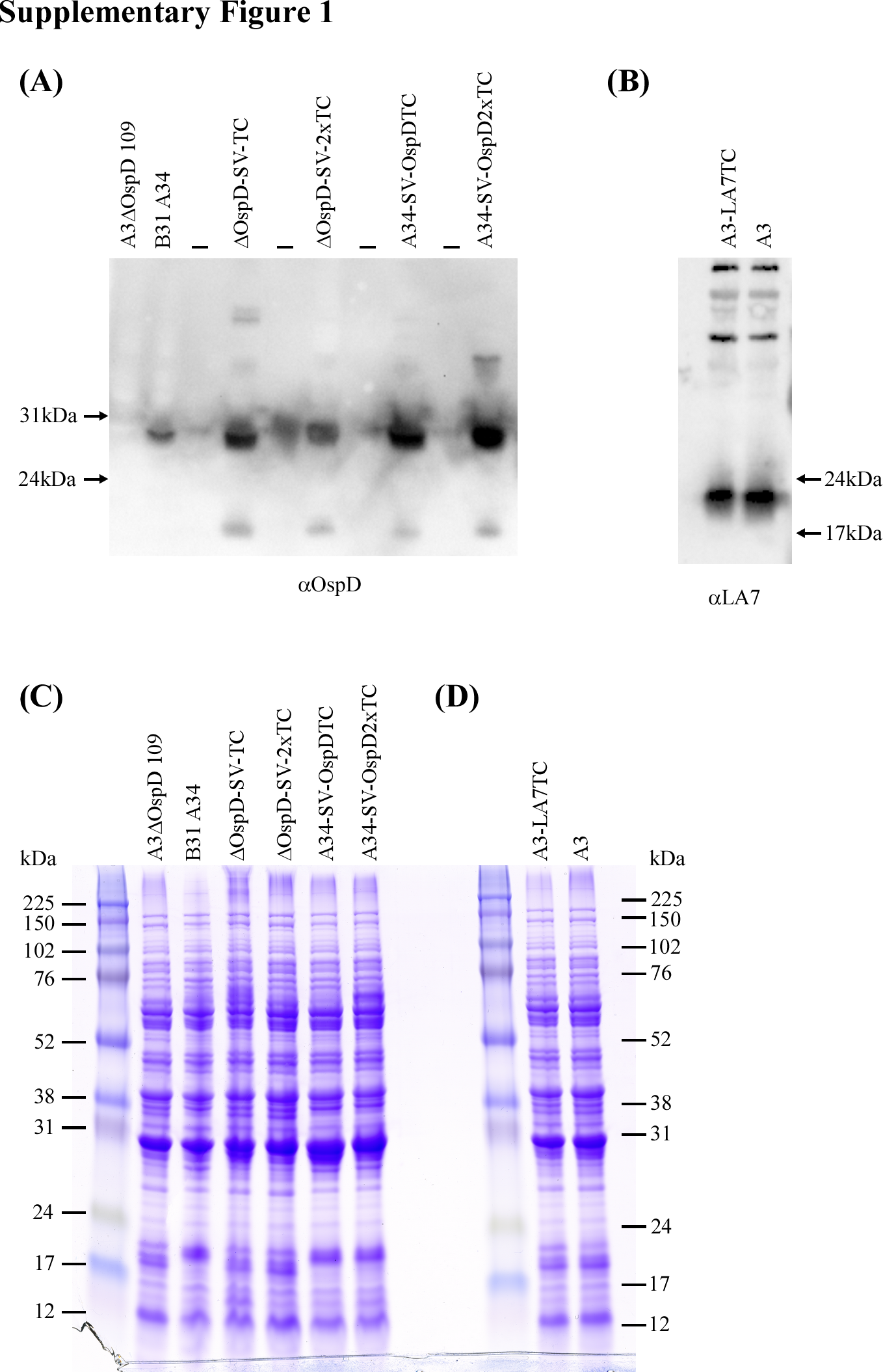

Supplement: Supplementary Figure 1 — OspD and LA7 protein levels in B31 strains. (A) Immunoblot analysis using antisera recognizing OspD (~28 kDa). OspD protein levels are comparable in strains transformed with SV-OspDTC and SV-OspD2xTC (B) Immunoblot analysis using antisera recognizing LA7 (~22 kDa). LA7 protein level is comparable between strains B31 A3 and A3-LA7TC. (C,D) Coomassie brilliant blue-stained polyacrylamide gel indicating relative protein loads of samples used for immunoblots in (A,B), respectively. [file Image_1.TIF]

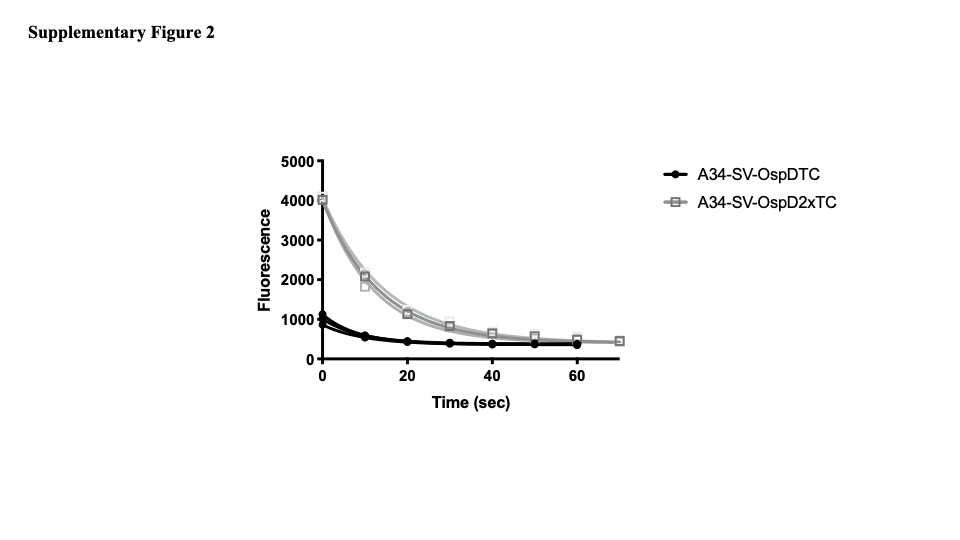

Supplement: Supplementary Figure 2 — Duration of fluorescence increased by the addition of a second tetracysteine motif. Fluorescence of strain A34-SV-OspDTC was compared to A34-SV-OspD2xTC over time. Videos were taken with Nikon Elements software and data analyzed in Prism. Measurements were taken of three spirochetes/strain during the same experiment. Fluorescence represents the intensity of spirochetes measured with the Nikon Elements software; time measured in seconds (s). [file Image_2.TIFF]

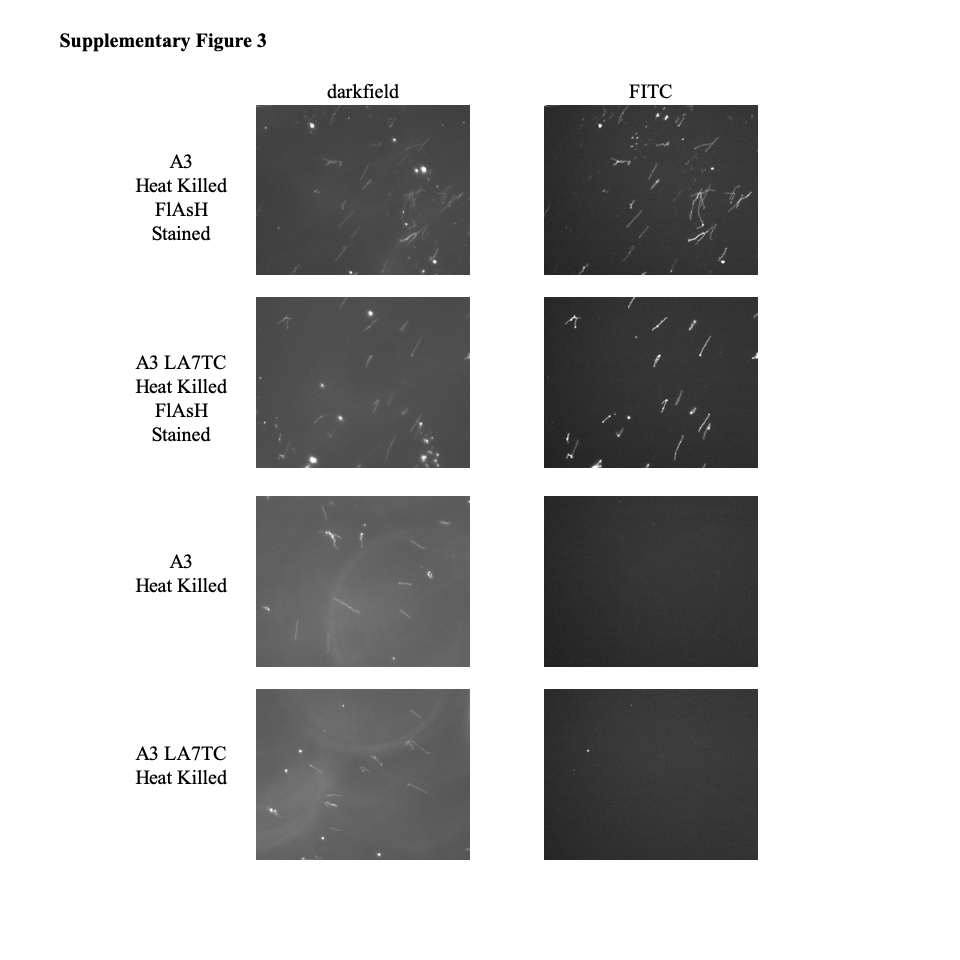

Supplement: Supplementary Figure 3 — Non-specific binding of FlAsH dye to heat-killed spirochetes. A3 spirochetes that do not contain a tetracysteine motif, but were heat killed prior to FlAsH staining, readily take up the dye and fluoresce (1st row) similarly to A3-LA7TC spirochetes containing a tetracysteine motif (2nd row). Spirochetes that have been heat-killed but not incubated with FlAsH, do not fluoresce (3rd and 4th row). Fluorescent spirochetes are only detected in the FITC channel, not in any other channels (not shown). [file Image_3.TIFF]

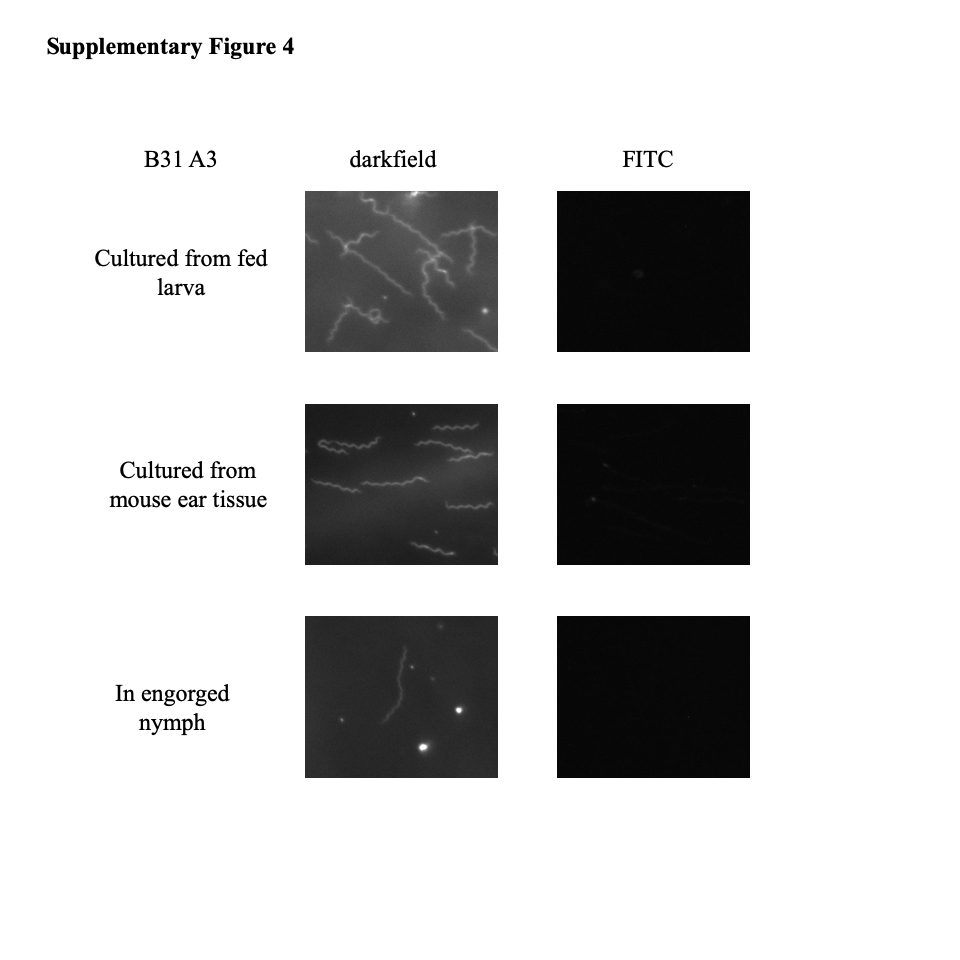

Supplement: Supplementary Figure 4 — B. burgdorferi wild-type spirochetes (B31 A3) isolated from tick vectors and murine hosts do not fluoresce after labeling. FlAsH-labeling of spirochetes isolated from larval ticks fed on B31 A3-infected mice (top); spirochetes isolated from ear tissue of mice fed on by B31-A3 infected nymphs (middle); and spirochete in dissected midgut of an engorged B31 A3-infected nymph (bottom). Representative dark field and FITC images are shown. [file Image_4.TIFF]
